# Expression of lactate-related signatures correlates with immunosuppressive microenvironment and prognostic prediction in ewing sarcoma

**DOI:** 10.3389/fgene.2022.965126

**Published:** 2022-08-24

**Authors:** Zhao Zhang, Jingxin Pan, Debin Cheng, Yubo Shi, Lei Wang, Zhenzhou Mi, Jun Fu, Huiren Tao, Hongbin Fan

**Affiliations:** ^1^ Department of Orthopaedic Surgery, Xi-jing Hospital, The Fourth Military Medical University, Xi’an, China; ^2^ Department of Orthopaedics, Shenzhen University General Hospital, Shenzhen, China

**Keywords:** Ewing sarcoma, lactate, prognostic prediction, immunosuppressive, nomogram

## Abstract

**Objectives:** Ewing sarcoma (EWS) is an aggressive tumor of bone and soft tissue. Growing evidence indicated lactate as a pivotal mediator of crosstalk between tumor energy metabolism and microenvironmental regulation. However, the contribution of lactate-related genes (LRGs) in EWS is still unclear.

**Methods:** We obtained the transcriptional data of EWS patients from the GEO database and identified differentially expressed-LRGs (DE-LRGs) between EWS patient samples and normal tissues. Unsupervised cluster analysis was utilized to recognize lactate modulation patterns based on the expression profile of DE-LRGs. Functional enrichment including GSEA and GSVA analysis was conducted to identify molecular signaling enriched in different subtypes. ESTIMATE, MCP and CIBERSORT algorithm was used to explore tumor immune microenvironment (TIME) between subtypes with different lactate modulation patterns. Then, lactate prognostic risk signature was built *via* univariate, LASSO and multivariate Cox analysis. Finally, we performed qPCR analysis to validate candidate gene expression.

**Result:** A total of 35 DE-LRGs were identified and functional enrichment analysis indicated that these LRGs were involved in mitochondrial function. Unsupervised cluster analysis divided EWS patients into two lactate modulation patterns and we revealed that patients with Cluster 1 pattern were linked to poor prognosis and high lactate secretion status. Moreover, TIME analysis indicated that the abundance of multiple immune infiltrating cells were dramatically elevated in Cluster 1 to Cluster 2, including CAFs, endothelial cells, Macrophages M2, etc., which might contribute to immunosuppressive microenvironment. We also noticed that expression of several immune checkpoint proteins were clearly increased in Cluster 1 to Cluster 2. Subsequently, seven genes were screened to construct LRGs prognostic signature and the performance of the resulting signature was validated in the validation cohort. Furthermore, a nomogram integrating LRGs signature and clinical characteristics was developed to predict effectively the 4, 6, and 8-year prognosis of EWS patients.

**Conclusion:** Our study revealed the role of LRGs in immunosuppressive microenvironment and predicting prognosis in EWS and provided a robust tool to predict the prognosis of EWS patients.

## Introduction

Ewing sarcoma (EWS) is the second leading malignant bone and soft tissue tumor, often arising in children and adolescents (Kaatsch et al., 2016). The highly aggressive and locally destructive feature of EWS accounts for early metastasis and local recurrence (Jin, 2020). As the clinical application of surgical procedures combined with chemotherapy and radiotherapy, the prognosis of EWS patients has improved remarkably (Fan et al., 2017; Zhu et al., 2021). Unfortunately, metastatic and recurrent patients are refractory to radiotherapy and chemotherapy, with the 5-year survival rates below 25% (Koustas et al., 2021). The emergence of resistance to conventional treatments has turned out to be a serious obstacle in the clinic. Hence, there is an emerging need for exploring novel therapeutic targets to improve the prognosis of EWS patients.

In 1926, Otto Warburg found that tumor cells do not rely on mitochondrial oxidative phosphorylation for energy supply under adequate oxygen conditions, rather they use aerobic glycolysis to produce large amounts of lactic acid to maintain tumor growth. This phenomenon is known as the Warburg effect (Warburg et al., 1927). Lactate, long considered as a waste product of glycolysis, has now proven to be a critical factor in the reprogramming of tumor metabolism and is strongly connected to tumorigenesis, immune microenvironment and therapeutic resistance (Baltazar et al., 2020). The sarcoma microenvironment is characterized by tumor interstitial acidification, increased lactate secretion can maintain microenvironmental acidification which participates directly in tumor development and metastasis (Taddei et al., 2020). Lactate has been shown to enter endothelial cells *via* the monocarboxylate transporter MCT-1 to activate the NF-κB/IL-8 pathway to facilitate tumor migration and vasculogenesis (Végran et al., 2011). In addition, lactate, as an immune-negative modulator, can regulate the differentiation of a variety of immune cells towards an immunosuppressive phenotype to promote tumor immune (Sangsuwan et al., 2020). Tumor cells are able to induce cancer-associated fibroblasts (CAFs) to generate hepatocyte growth factor *via* increased lactate secretion, thereby activating MET-dependent signaling to trigger drug resistance (Apicella et al., 2018). These findings indicated that targeting lactate metabolism could be a prospective treatment strategy for tumors. Previous studies have identified that lactate dehydrogenase levels are tightly related to prognosis and treatment response in EWS patients (Fu et al., 2016; Li et al., 2016). Given the pivotal effect of lactate in tumorigenesis and immunosuppression, comprehensive analysis of lactate-related genes (LRGs) to reveal their potential role in EWS is important.

Recently, as high-throughput sequencing has evolved, stratifying tumor patients by bioinformatics and machine learning approaches to explore new biomarkers has proven to be reliable and useful (Zhang et al., 2022a; Zhang et al., 2022b; Sun et al., 2022). In the current study, we first screened differential expression ofLRGs (DE-LRGs) between EWS and normal tissues. Then, we identified two lactate modulation patterns derived from the expression profile of DE-LRGs and evaluated the prognosis and tumor immune microenvironment (TIME) of the two different subtypes. Moreover, a novel prognostic signature constructed by seven candidate LRGs accurately predicted the prognosis of EWS patients. These findings identified potential new biomarkers that can be used for clinical decision making in EWS patients.

## Methods and materials

### Data collection

The transcriptional and clinical data on Ewing sarcoma patients were acquired from the GSE17679 dataset in the GEO database as a training cohort, which included 88 EWS tissues and 18 normal tissues (Barrett et al., 2013). The word “lactic” was used as a keyword to extract LRGs from the Molecular Signatures Database (MSigDB; https://www.gsea-msigdb.org/gsea/msigdb/index.jsp) (Sun et al., 2022) (Supplementary Table S1). After removing duplicates, a total of 252 genes were evaluated in this study. In addition, the transcriptomic and clinical data of 56 EWS patients were obtained from the International Cancer Genome Consortium (ICGC) database as an external independent validation cohort (Hudson et al., 2010). The clinical information in the both cohorts was shown in Supplementary Table S2.

### Identification of differential expression ofLRGs in the training cohort

The R package “limma” was employed to identify DE-LRGs between EWS and normal tissues, and adj. *p* < 0.05 and |log2 fold change (FC)| ≥ 1.50 was set as the threshold. Heat map was applied to present the expression of DE-LRGs in tumor and normal tissue. Subsequently, Gene Ontology (GO) term and Kyoto Encyclopedia of Genes and Genomes (KEGG) pathway were applied to assess the enrichment of functional pathways within these DE-LRGs.

### Unsupervised clustering analysis

Unsupervised clustering analysis was performed to determine lactate modulation patterns based on the expression profile of DE-LRGs through the R package “ConsensusClusterPlus”, with the following conditions to ensure stability:maxK = 6, reps = 1000, pItem = 0.8, pFeature = 1, clusterAlg = hc and distance = pearson. The cumulative distribution function (CDF), consensus matrix, and comparative change in area under the CDF curve were taken to ensure the optimal number of clusters. Kaplan Meier (KM) curves were utilized to assess overall survival (OS) between different subtypes. Subsequently, Gene Set Enrichment Analysis (GSEA) and Gene Set Variation Analysis (GSVA) were performed to assess the hallmark gene sets in different subtypes.

### Tumor immune microenvironment analysis of lactate modulation patterns

To further characterize the TIME of the different subtypes, the ESTIMATE algorithm was employed to calculate the TIME scores for each sample, and t-test to compare the differences between subtypes (Yoshihara et al., 2013). Subsequently, the MCP counter and CIBERSORT algorithm was used to assess the abundance of different immune infiltrating cells in different subtypes. Furthermore, we also evaluated the differences of expression of immune checkpoints (ICPs) proteins in different subtypes.

### Establishment and validation of LRG prognostic signature

To appraise the prognostic role of LRGs, univariate Cox analysis was applied to screen for DE-LRGs, *p* < 0.05 was deemed prognosis-related LRGs. The least absolute shrinkage and selection operator (LASSO) Cox regression was utilized to prevent overfitting of the prognostic model by R package “glmnet”, and the minimum lamba value was adopted as the optimal value. Finally, multivariate Cox regression was employed to build prognostic signature. The risk score in each patients was calculated based on the following formula:Risk score = Σin(Coefi * Xi). Based on risk scores, patients in the training cohort were classified into high-risk and low-risk groups, and KM curve and time-dependent ROC curves were availed to assess the prognostic performance of the model. Moreover, the validation cohort was performed to verify the accuracy of the LRGs signature by the above formula. In addition, combining risk score and clinical characteristics, Cox regression was applied to estimate whether risk score was an independent prognostic element for EWS patients.

### Quantitative real-time PCR

The human EWS cell line A673 and RD-ES were obtained from Procell Life Science Technology Co.,Ltd. and Meisen Cell Technology Co., Ltd., respectively. The hMSC cells were purchased from Hengya Biotechnology Co., Ltd. as normal control. A673 and hMSC were cultured in DMEM medium containing 10% fetal bovine serum (FBS) and 1% (v/v) P/S. RD-ES was cultured in RPMI 1640 medium containing 10% FBS and 1% (v/v) P/S. All cells were incubated at t 37°C, 5% CO_2_. Subsequently, the TRIZOL method was used to extract and purify RNA from cells and cDNA synthesis kit (Takara, China) were utilized to reverse transcribe the RNA. The TB Green Premux Ex Taq II (Tli RNaseH Plus) and Bio-Rad CFX96 real-time PCR system (Bio-Rad, United States) was employed for qRT-PCR. The internal control was GADPH. The primer sequences of the candidate genes are shown in Table 1, and all genes were repeated three times for analysis.

**TABLE 1:** The primer sequences of the candidate genes.

Gene	Sequence (5′ -> 3′)
PUS1	Forward:GTCTGGGAGGACGGAGAACAT
	Reverse:CAGCACGATCTTCCGCTTG
NDUFB9	Forward:GTGGTGCGTCCAGAGAGAC
	Reverse:GGCCTTCGCCATATCCTTTTC
NDUFB10	Forward:AGCCCAATCCCATCGTCTACA
	Reverse:GCTGCCGCTCTATAAATTCTCT
SLC25A12	Forward:TCAAGGTGCAGACAACTAAGC
	Reverse:GGGGTCATATAACGCTCTCCA
COX6A2	Forward:CCTTCAACTCCTATCTCCACTCG
	Reverse:GTTGGTAGGGACGGAACTCG
PPM1B	Forward:TGGGAATGGTTTACGTTATGGC
	Reverse:GCCGTGAGGAATACCTACAACAG
RYR1	Forward:GACAGGGAACACGACCACTATTA
	Reverse:ATGACATCCTTGCCCGAGTAGTA
GADPH	Forward:GGAGCGAGATCCCTCCAAAAT
	Reverse:GGCTGTTGTCATACTTCTCATGG

### Construction and calibration of nomogram

A nomogram model was developed to predict the probability of survival of EWS patients by integrating risk scores and clinical characteristics. The C-index, calibration plots and ROC curves were adopted to measure the predictive performance of the constructed nomogram at 4, 6, and 8 -year.

### Statistics

All statistical analyses were conducted using R 4.0.5 software, GraphPad Prism 8 and SPSS 21.0. The t-test was performed to two groups, and one-way ANOVA was performed to three groups. *p* < 0.05 was deemed to a statistical difference. *p < 0.05; **p < 0.01; ***p < 0.001.

## Result

### Identification and functional enrichment of differential expression ofLRGs

**FIGURE 1:**
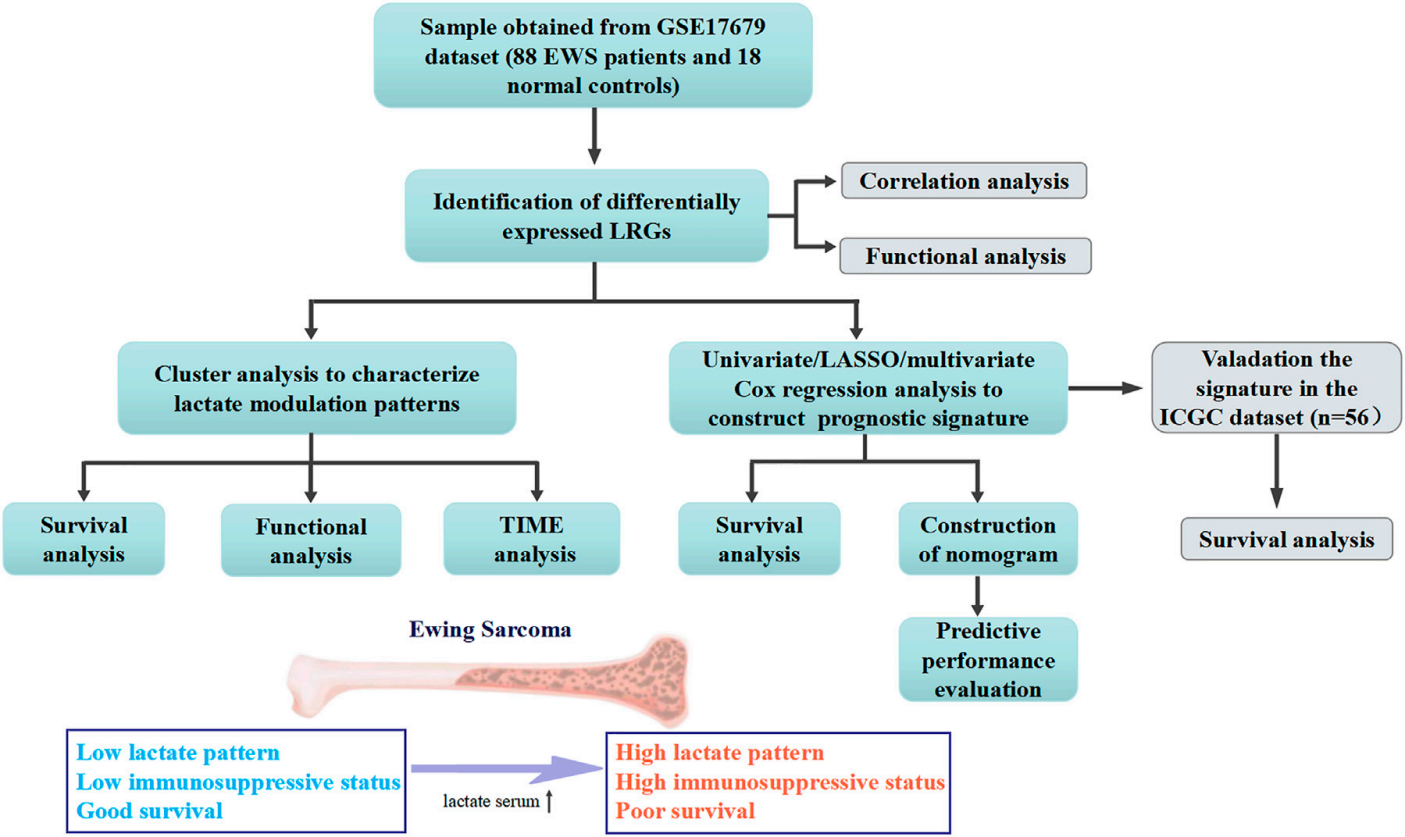
Workflow diagraph of the data analyzing process.

**FIGURE 2:**
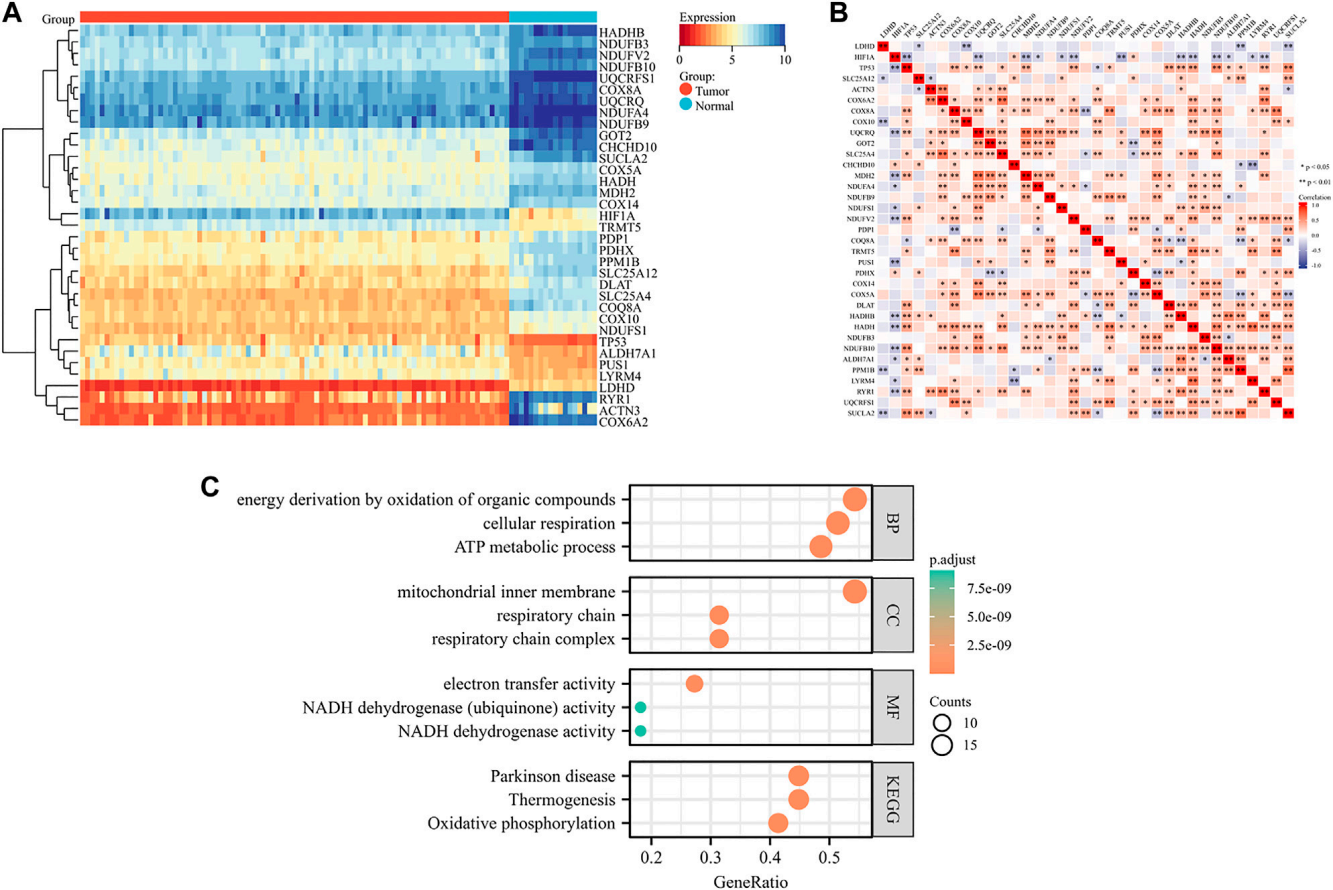
Identification and functional enrichment of DE-LRGs in EWS **(A)**. Heat map illustrating the expression of DE-LRGs in each sample **(B)**. Interaction plot of DE-LRGs in EWS **(C)**. GO terms and KEGG pathway analysis.

The workflow of this study was shown in Figure 1. We identified a total of 35 DE-LRGs between EWS and normal tissues, of which six genes were up-regulated and 29 genes were down-regulated in expression. Heat map presenting the expression levels of DE-LRGs in EWS and normal tissues (Figure 2A). Then, we assessed the interaction of these genes in the EWS (Figure 2B). Moreover, KEGG analysis indicated that these DEGs were mainly enriched in thermogenesis, oxidative phosphorylation and Parkinson disease. GO terms indicate that these DEGs were primarily involved in ATP metabolic process, energy derivation by oxidation of organic compounds, cellular respiration amongst biological process (BP); were primarily involved in respiratory chain, respiratory chain complex and mitochondrial inner membrane amongst cellular component (CC), were primarily involved in NADH dehydrogenase (ubiquinone) activity, NADH dehydrogenase activity and electron transfer activity amongst molecular function (MF) (Figure 2C).

### Identification of lactate modulation patterns by clustering analysis

**FIGURE 3:**
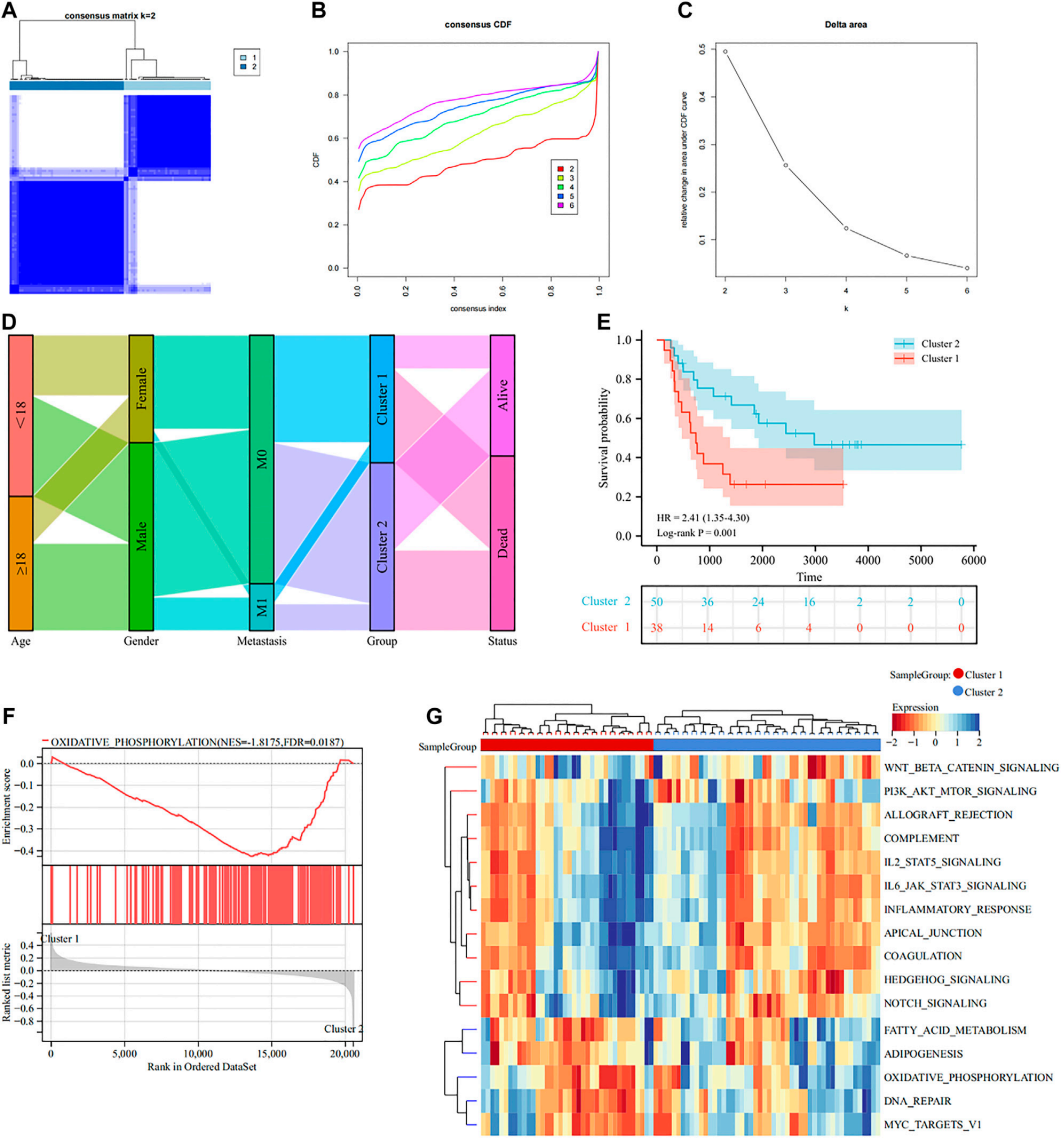
Classification of lactate modulation patterns by clustering analysis **(A–C)**. K = 2 was regarded as the optimal number of subtype clusters **(D)**. Sankey diagram showing the association of different patterns with clinical features **(E)**. KM curve presenting the prognosis of different patterns **(F)**. GSEA analysis showcasing different patterns of hallmark gene sets **(G)**. GSVA analysis exhibiting the functional enrichment of different patterns.

To understand the role of lactate modulation patterns in EWS, we classified EWS patients into different subtypes through unsupervised clustering analysis using the expression profiles of DE-LRGs, and k = 2 was identified as the optimal number of clusters (Figures 3A–C). Sankey diagram illustrated the relationship between different patterns and clinical features and survival status (Figure 3D). Moreover, KM curve indicated that patients with Cluster 1 had a noticeably poor prognosis compared to Cluster 2 (Figure 3E). GSEA analysis revealed that the oxidative phosphorylation was significantly enriched in Cluster 2 amongst hallmark gene sets (Figure 3F). Besides, GSVA analysis found that multiple classical tumor-related and immune-related pathways enriched in Cluster 1, including WNT beta catenin signaling, IL6/JAK/STAT3 signaling, inflammatory response, hedgehog signaling etc, whereas oxidative phosphorylation were significantly upregulated in Cluster 2. These findings indicated that different biological functions might be intimately linked to the prognosis of lactate modulation patterns (Figure 3G).

### Tumor immune microenvironment analysis of lactate modulation patterns

**FIGURE 4:**
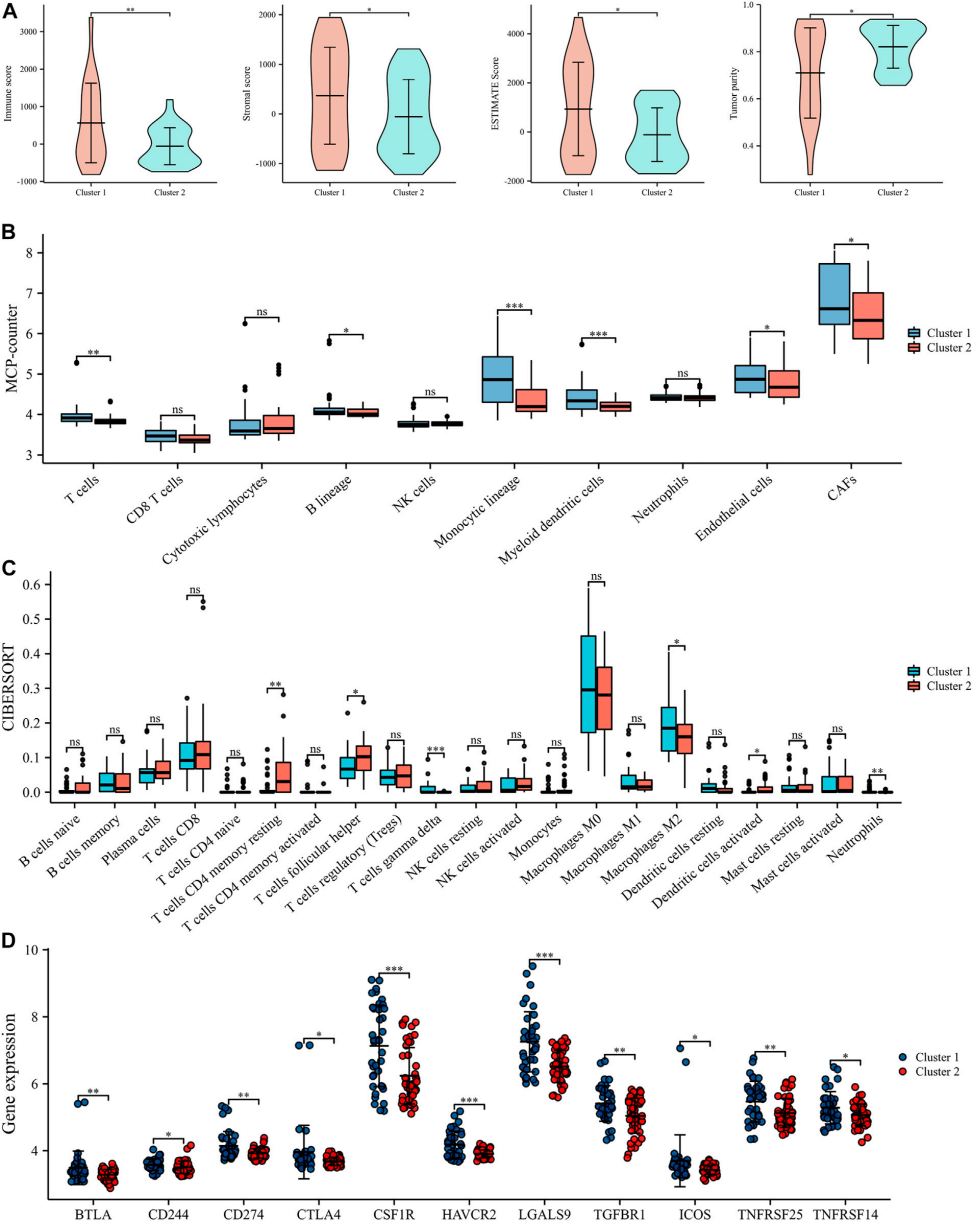
Tumor immune microenvironment (TIME) evaluation in different lactate regulation patterns **(A)**. TIME scores by ESTIMATE algorithm **(B)**. The expression levels of 10 immune infiltrating cells by MCP counter **(C)**. Abundance of 22 immune infiltrating cells by CIBORSORT **(D)**. The expression levels of ICPs in different lactate regulation patterns. *p < 0.05; **p < 0.01; ***p < 0.001.

Substantial studies prove that lactate has a major impact on immune regulation in TIME, thus we carried out immune analysis to uncover the action of TIME in different subtypes. ESTIMATE algorithm indicated that Cluster 1 had better immune score (*p* = 8.4e-5), stromal score (*p* = 8.1e-7), ESTIMATE score (*p* = 9.6e-7) and worse tumor purity (*p* = 1.1e-6) than Cluster 2 (Figure 4A). MCP algorithm found that CAFs, Endothelial cells, Myeloid dendritic cells, Monocytic lineage, B lineage and T cells were significantly elevated in Cluster 1 to Cluster 2 (Figure 4B). CIBERSORT algorithm illustrated that the infiltration abundance of T cells gamma delta and Macrophages M2 were clearly elevated in Cluster 1 than in Cluster 2, whereas T cells CD4 memory resting, T cells follicular helper and Neutrophils showed the opposite result (Figure 4C). In addition, we also noted that the expression of some ICPs was clearly elevated in Cluster 1 over Cluster 2, which suggested that Cluster 1 appeared to be intimately associated with the immunosuppressive microenvironment (Figure 4D). Altogether, these results indicate that different LRGs subtypes might influence the progression, invasion and prognosis of EWS through modulating TIME.

### Development and validation of the LRGs prognostic signature

**FIGURE 5:**
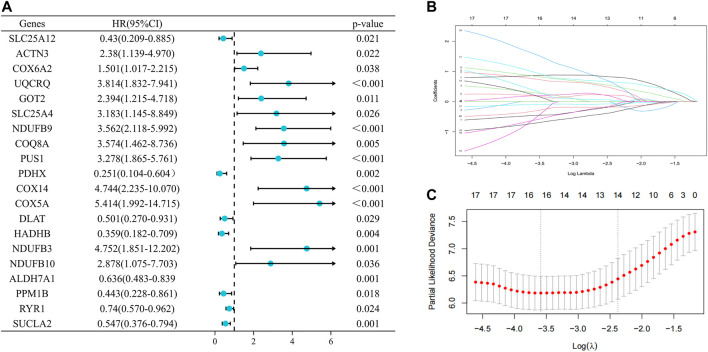
Selection of prognosis-related DE-LRGs to construct prognostic models **(A)**. The forest plot present the DE-LRGs associated with prognosis *via* univariate Cox analysis **(B ,C)**. Lasso analysis further screened 14 genes with the optimal lambo value.

**FIGURE 6:**
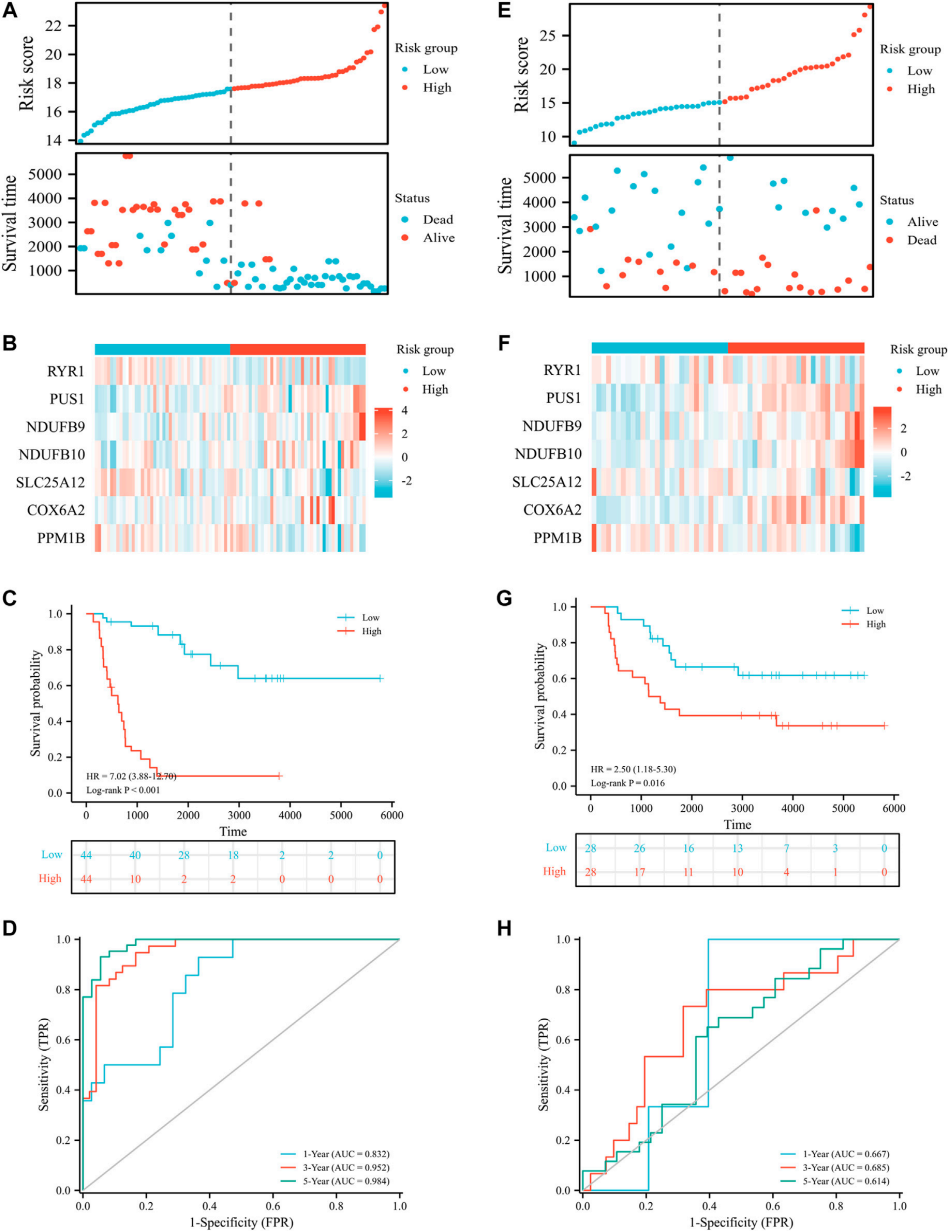
Prognostic value of LRGs signature in EWS patients. Distribution plots of the risk score and survival status in the training **(A)** and validation cohort **(E)**; Heat map showing the expression of candidate genes for different risk groups in the training **(B)** and validation cohort **(F)**; The survival analysis in the training **(C)** and validation cohort **(G)**; The ROC curve for the training **(D)** and validation cohort **(H)**.

To assess the prognostic value of LRGs in EWS patients, 20 prognostically correlated DE-LRGs were identified by univariate Cox analysis (Figure 5A). Then, LASSO analysis was employed to further filter prognostic genes and 14 LRGs were confirmed by minimal lambo value (Figures 5B,C). Finally, seven candidate genes (PUS1, COX6A2, NDUFB9, NDUFB10, SLC25A12, PPM1B and RYR1) were identified by multivariate Cox analysis to construct a prognostic signature. The following formula was adopted to calculate the LRGs score for each sample. LRGs score = 1.095 × PUS1 + 0.827× COX6A2 + 0.883 × NDUFB9 + 1.943 ×NDUFB10 – 0.887× SLC25A12 – 1.311 × PPM1B – 0.884 × RYR1. Subsequently, patients in the training cohort were separated into high and low risk groups depending on the median of the LRGs score. As presented in Figures 6A,B, the survival time of EWS patients decreased with increasing LRGs scores. KM curves demonstrated that the high-risk group had a worse prognosis than the low-risk group (Figure 6C). The ROC curves suggested that the LRGs prognostic signature had AUC values of 0.832, 0.952, and 0.984 at 1, 3, and 5 years, respectively, which indicated that the model exhibited excellent predictive accuracy (Figure 6D).

**FIGURE 7:**
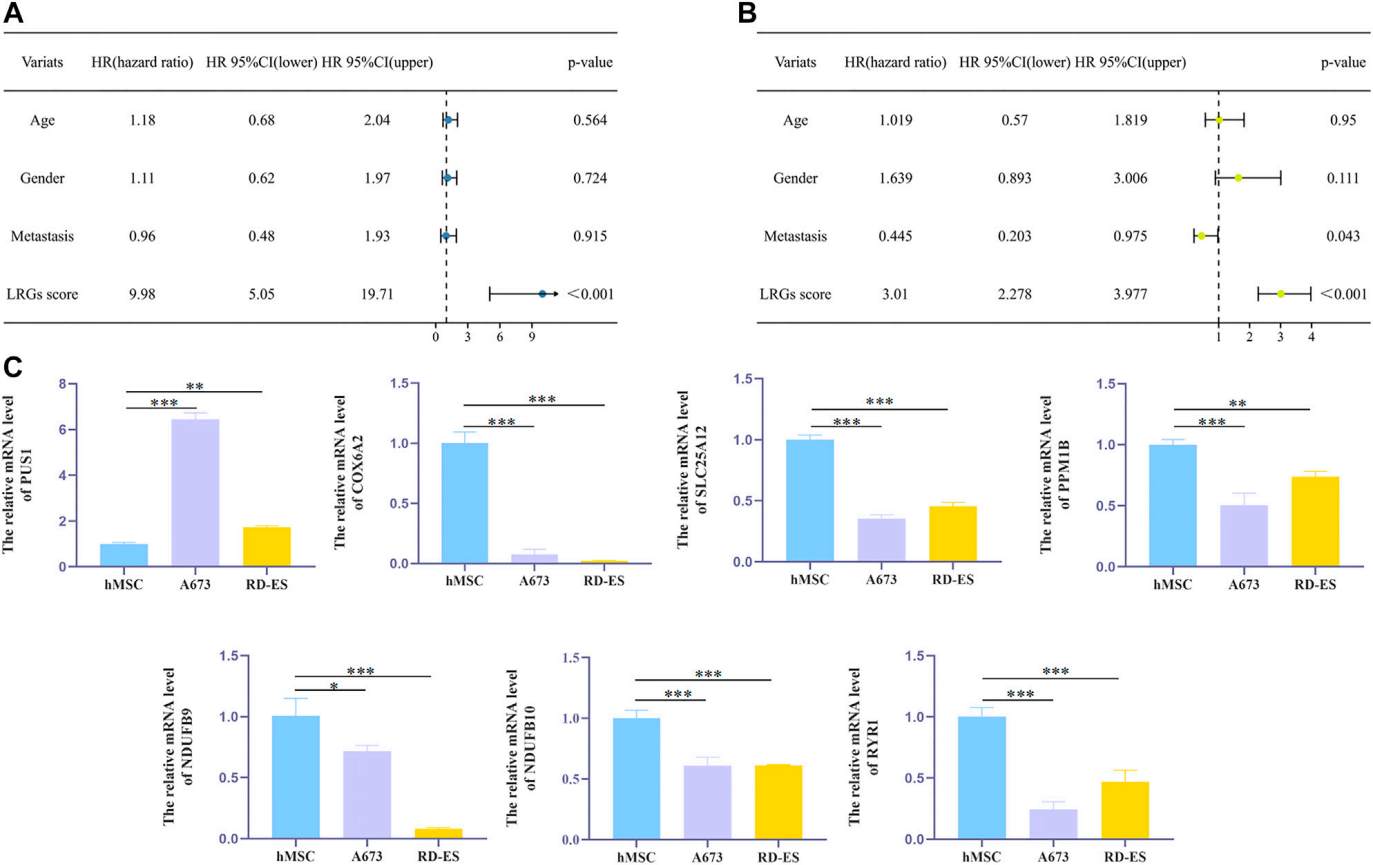
The effect of independent factors on LRGs scores and qPCR analysis **(A,B)**. Univariate and multivariate Cox analyses *via* integrating LRGs scores and clinical characteristics in the training cohort **(C)**. qPCR analysis to verify the expression level of candidate genes in cells. *p < 0.05; **p < 0.01; ***p < 0.001.

Then, patients in the validation cohort were divided into high and low risk groups for validation of the LRGs score signature based on the median LRGs score. Consistent with the results of the training cohort, we observed that patients in the low-risk group exhibited longer survival and improved general survival compared to the high-risk group (Figures 6E–G). ROC curves revealed that the 1, 3, and 5-year AUC values were 0.667, 0.685, and 0.614, respectively (Figure 6H). By integrating LRGs score and clinical feathers, multivariate Cox analysis identified LRGs score as independent prognostic factors for patients with EWS in the training cohort by integrating LRGs score and clinical feathers (Figures 7A,B). Moreover, we validated the expression of these candidate genes between EWS cell lines and hMSC. The results showed that PUS1 was significantly upregulated in EWS cell lines, while NDUFB9, NDUFB10, SLC25A12, COX6A2, RYR1 and PPM1B were significantly downregulated in EWS cell lines than the hMSC(Figure 7C).

### Construction and validation of a nomogram for predicting prognosis

**FIGURE 8:**
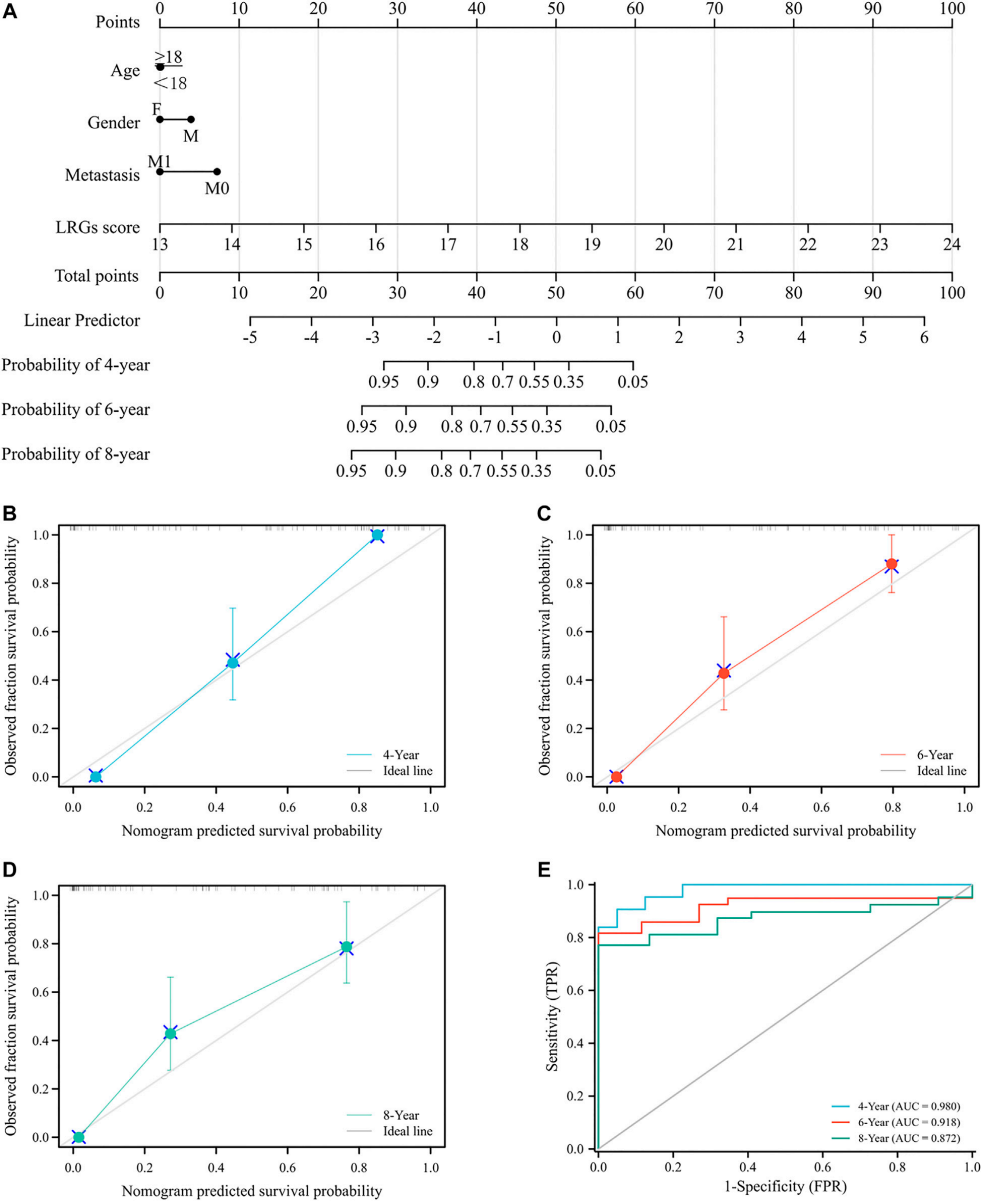
Construction and evaluation a novel nomogram for EWS patients **(A)**. Constructed nomogram to predict the 4, 6 and 8-year survival rate for EWS patients **(B–D)**. Calibration curve to verify the accuracy of the nomogram **(E)**. ROC curve of the nomogram.

To assess the utility of LRGs scores in predicting EWS prognosis, we integrated LRGs scores and clinical feathers to construct a nomogram model in the training cohort (Figure 8A). The C-index of the nomogram was 0.846, which suggested that the constructed nomogram had excellent predictive value. Calibration curve showed that the predicted outcomes had high consistency with the actual outcomes (Figures 8B–D). The ROC curves had AUC values of 0.980, 0.918 and 0.872 for 4, 6, and 8-year, respectively (Figure 8E). Collectively, these results showed that constructed nomogram had robust predictive accuracy for the prognosis of EWS patients.

## Discussion

Glucose metabolism shifts from oxidative phosphorylation to aerobic glycolysis in order to generate large amounts of lactate to fuel tumor growth is an essential feature of tumor metabolic reprogramming (Lu et al., 2015). Lactate is a pivotal mediator of crosstalk between tumor metabolism and microenvironment which is tightly linked to angiogenesis, immune escape and tumor metastasis (Baltazar et al., 2020). Previous studies have proven that increased serum lactate dehydrogenase in EWS patients was strongly associated with poor prognosis (Li et al., 2016). Yeung et al. (2019) found that targeting lactate dehydrogenase could modulate the transcription of EWS-FLI1, an oncogenic driver of EWS, to inhibit the tumor proliferation and induce cell apoptosis. In addition, lactate dehydrogenase activity was shown to be a marker for the efficacy of chemotherapy and radiotherapy in EWS patients (Fu et al., 2016; Shapiro et al., 2018). These results point to an essential contribution of lactate metabolism in EWS. Nonetheless, the immunological and prognostic role of LRGs in EWS patients remains unclear.

In the present study, we identified 35 DE-LRGs by comparing transcriptome data from EWS and normal tissues, most of which were significantly downregulated in EWS patients. GO and KEGG analysis showed that these DE-LRGs were enriched in oxidative phosphorylation, electron transfer activity, mitochondrial inner membrane, and cellular respiration pathways, which were closely related to aerobic respiration. Cancer cells are known to transform glucose into lactate through the Warburg Effect presence of oxygen, thereby supplying energy for tumor growth and proliferation (Lu et al., 2015). We speculated that the downregulation of the expression of LRGs in EWS could contribute to mitochondrial dysfunction and inhibit mitochondrial respiration in tumor cells, which would in turn facilitate the shift from oxidative phosphorylation to aerobic glycolysis resulting in increased lactate secretion. These findings provided new insights into the development and progression of EWS.

Stratification of tumor patients to personalize therapy can significantly improve patient prognosis (Martinez-Useros et al., 2021). Our study identified two lactate modulation patterns by unsupervised clustering analysis of the expression profiles of DE-LRGs. KM curve revealed that patients with Cluster 1 had a substantially worse prognosis than those with Cluster 2. Tumor metabolic reprogramming, an essential feature of tumor progression, would result in decreased oxidative phosphorylation and increased lactate generation to match the energy demand of rapid tumor growth. GSEA analysis revealed that oxidative phosphorylation was upregulated in Cluster 2, indicating that Cluster 2 might be associated with low lactate secretion pattern, while Cluster 1 might be associated with high lactate secretion pattern. Mizushima et al. (2020) already demonstrated that the metabolism of high oncogenic osteosarcoma cell lines were dependent on lactate production from aerobic glycolysis and inhibition of oxidative phosphorylation as compared to low oncogenic ones. Further analysis identified Cluster 1 to be linked to immune regulation, hedgehog signaling, notch signaling and etc, while Cluster 2 was linked to oxidative phosphorylation, which further validated our results. Marino et al. (2014) demonstrated that elevated lactate dehydrogenase was an independent poor prognostic factor in patients with EWS. Tural et al. (2012) similarly found that poor prognosis in patients with extraskeletal EWS was tightly coupled with elevated lactate dehydrogenase. Therefore, we think that the adverse prognosis of EWS patients in Cluster 1 might be linked to high lactate secretion pattern.

The tumor microenvironment has a variety of components, among which lactate serves as a critical mediator of tumor metabolism and can drive tumor progression by regulating TIME (Baltazar et al., 2020). We evaluated TIME of both lactate patterns and the results demonstrated that the immune score and stromal score were clearly increased in Cluster 1 than in Cluster 2. Our finding is consistent with the fact that immune cells and/or stromal cells have been shown to be an important source of lactate secretion in TIME. Immune infiltrating cell analysis revealed a significant rise in the infiltration abundance of a variety of immune cells in Cluster 1, including CAFs, endothelial cells, Macrophages M2, and others. The elevated levels of lactate in the TIME can regulate the immune response, angiogenesis and cell invasion to promote tumor growth (Romero-Garcia et al., 2016). Bhagat et al. (2019) found that tumor cell-secreted lactate could promote the formation of CAFs by regulating epigenomic reprogramming, resulting in enhanced the invasiveness of tumor. Sonveaux et al. (2012) showed that lactate could activate HIF-1 signaling in endothelial cells to induce tumor angiogenesis. Vadevoo et al. (2021) identified that lactate could drive macrophage M2 polarization through the odor receptor Olfr78 to promote immune escape and tumor progression. Besides, hypoxic tumor environment could cause a decrease in miR-34a to increase tumor lactate levels which trigger the dysfunction of immune cell (Ping et al., 2018). Extensive researches have demonstrated that lactate is a critical factor in tumor immune escape which could promote tumorigenesis and progression by interfering with stromal/immune cells to create an immunosuppressive microenvironment (Romero-Garcia et al., 2016). Furthermore, we found that several ICPs were expressed higher in Cluster1 than Cluster2, such as CD274 and CTLA4, which are currently broadly available in the clinic. Chen et al. (2021) found that lactate could alter lytic granule cytosolic interaction to induce CD8^+^ T cell dysfunction, leading to a sustained increase in CD274 expression. Recently, a multicenter retrospective cohort study found that combined anti-PD-1 and anti-CTLA-4 blockade markedly improved survival time in BRAF-mutated melanoma patients with increased lactic dehydrogenase, which suggested that increased lactate secretion could significantly increase the expression of ICPs contributing to immune escape in the TIME (Knispel et al., 2021). Neutralization the lactate levels in the EWS microenvironment might suppress immune checkpoints to boost anti-tumor immunity and thus effectively improve the prognosis of EWS patients. Collectively, these findings revealed that lactate levels were closely linked to the immunosuppressive microenvironment of EWS, presenting a promising targeting strategy for the treatment of EWS patients.

To further explore the prognostic role of these LRGs in EWS, we used univariate, LASSO and multivariate Cox analyses to screen candidate genes. Ultimately, 7 LRGs were identified to construct prognostic models, with PUS1, NDUFB9, NDUFB10 and COX6A2 as risk factor and SLC25A12, RYR1 and PPM1B as a protective factor for the prognostic of EWS patients. PUS1 encodes a pseudouridine synthase which serves an essential function in the structural modification of tRNA and mRNA. Numerous researches revealed that aberrant expression of PUS1 was closely linked to lactic acidosis and mitochondrial myopathy (Knispel et al., 2021). Li et al. (2021) suggested that elevated levels of PUS1 were intimately connected with poor prognosis of hepatocellular. NDUFB9 and NDUFB10 were associated with mitochondrial respiratory chain complex I assembly. Li et al. (2021) found that deletion of NDUFB9 promoted proliferation and invasion of metastatic breast cancer cells *via* Akt/mTOR/p70S6K axis. Hebert-Chatelain et al. (2012) revealed that activation of Src kinase could inhibit NDUFB10 phosphorylation and decrease mitochondrial respiration to increase aerobic glycolysis. COX6A2 is implicated in the formation of the cytochrome c oxidase (COX) subunit, which are the terminal enzymes of the mitochondrial respiratory chain regulating electron transfer. Inoue et al. (2019) found that mutations in COX6A2 could lead to reduced activity of mitochondrial respiratory chain complexes IV which resulted in defective mitochondrial respiratory chains. SLC25A12 can encode a calcium-binding mitochondrial carrier protein, which is responsible for the conversion of aspartate to glutamate in the inner mitochondrial membrane. SLC25A12 deletion could affect mitochondrial respiration and NAD+/NADH ratio to increase lactate secretion in favor of tumor growth and metastasis (Alkan et al., 2020; Pérez-Liébana et al., 2022). RYR1 is a lanosteric receptor in skeletal muscle which mediates normal muscle contraction through the release of Ca2+. Yu et al. (2019) showed that the mutation of RYR1 was strongly connected with a favorable prognosis of immunotherapy for lung cancer. Previous studies demonstrated a significant increase in oxidative stress levels in patients with bone metastatic cancer, which decreased Ca2+-induced muscle contraction contributing to muscle weakness (Waning et al., 2015). PPM1B, a member of the PPM family of Ser/Thr protein phosphatases, acts as a negative regulator of the cellular stress response. Miller et al. (2018) found that depletion of PPM1B could activate the p38-RB1-E2F1 pathway to increase chemotherapy sensitivity to trigger tumor cell death. Yang et al. (2016) revealed that overexpression of PPM1B dramatically inhibited the proliferation and invasion of bladder cancer, which might be a new therapeutic target for bladder cancer. In our study, we demonstrated that the LRGs prognostic model had excellent predictive performance in both the training and validation cohort through KM and ROC curve analyses. Patients with high LRGs score were associated with with poor prognosis. In addition, our results also revealed that the LRGs prognostic model could be used as an independent predictor of prognosis in EWS patients. Lastly, we integrated the LRGs score and clinical characteristics to construct a novel nomogram which could accurately predict the prognosis of EWS patients at 4, 6 and 8 years, providing a new method to clinical decisions in EWS patients.

This study present the first comprehensive analysis of the role of lactate-related genes in EWS with many strengths, but there are some limitations. First, the specific mechanisms of lactate regulation of EWS progression remain unclear, an investigation of candidate genes is needed to unravel these processes at a later stage. Second, our study is a retrospective analysis based on publicly available databases lacking pathological information related to prognosis, which maybe biased. A large-scale multi-center prospective studies are required to further corroborate the clinical value of the above findings.

## Conclusion

In conclusion, we identified for the first time DE-LRGs involved in the development of EWS and investigated the role of lactate modulation patterns in EWS. The high lactate patterns could shape the immunosuppressive microenvironment contributing to the poor prognosis of EWS patients. Meanwhile, a novel LRGs prognostic signature was developed and validated to accurately predict the prognosis of EWS patients. Together, these findings shed light on the role of LRGs in the progression and prognosis of EWS and provide new insights for targeted therapy as well as prognosis prediction of EWS patients.

## Data Availability

The original contributions presented in the study are included in the article/Supplementary Material, further inquiries can be directed to the corresponding author.
